# Hypertension, uncontrolled hypertension and resistant hypertension: prevalence, comorbidities and prescribed medications in 228,406 adults resident in urban areas. A population-based observational study

**DOI:** 10.1007/s11739-023-03376-8

**Published:** 2023-08-02

**Authors:** Simone Romano, Giulio Rigon, Martina Albrigi, Giacomo Tebaldi, Andrea Sartorio, Luca Cristin, Giulia Burrei, Cristiano Fava, Pietro Minuz

**Affiliations:** 1 Section of Internal Medicine C, Department of Medicine, University of Verona, Verona, Italy; 2 FIMMG Study Centre Verona, Verona, Italy

**Keywords:** Hypertension, Antihypertensive therapy, Blood pressure control, Resistant hypertension, Uncontrolled hypertension, Population

## Abstract

Although hypertension is the leading cause of cardiovascular disease and premature death worldwide, it remains difficult to control. The prevalence of uncontrolled and resistant hypertension (RH) may be underestimated and can reach up to 50% of all hypertensive patients. The aim of this observational study was to analyze the prevalence of hypertension, uncontrolled hypertension and RH, and their associations with risk factors or diseases in a large cohort of patients referred to primary care physician. In a population of 228406 adults, we only collected data from people with a diagnosis of arterial hypertension for a total of 43,526 patients. For this purpose, we used the MySQL database, run by Azalea.NET, built on the medical records of 150 General Practitioners (GPs). Patient data included sex, age, blood pressure (BP) values, number of antihypertensive drugs and presence of major cardiovascular comorbidities. We classified patients with RH as those treated with 3 different antihypertensive agents, with recorded BP ≥ 140/90 mmHg, or patients taking ≥ 4 medications. The prevalence of hypertension was 19.06%, that of resistant hypertension was 2.46% of the whole population and 20.85% of the hypertensive group. Thirteen thousand hundred, forty-six patients (30.20% of the hypertensive group) had uncontrolled BP (≥ 140/90 mmHg), whereas 16,577 patients did not have BP measurements done in the last 2 years (38.09% of the hypertensive group). Patients with uncontrolled BP were mainly female, used less drugs and showed a lower prevalence of all major cardiovascular comorbidities, except for diabetes. Instead, patients with RH had a significantly higher prevalence of all considered comorbidities compared to those without RH. Our results evidence that a broad number of patients with hypertension, especially those without comorbidities or with a low number of antihypertensive drugs, do not achieve adequate BP control. To improve the clinical management of these patients it is very important to increase the collaboration between GPs and clinical specialists of hypertension.

## Introduction

Hypertension is the leading cause of cardiovascular disease and premature death worldwide [1]. Uncontrolled blood pressure (BP) is defined by levels ≥ 140/90 mmHg. This is a widespread pathology; according to a 2010 survey, the worldwide prevalence among adults is 31.1% (1.39 billion), and it is higher in low/medium-income countries [2]. Analyzing the situation in Italy, some difference among studies does emerge [1]. For instance, one study reports hypertension prevalence of 25.9%, while in another recent is, respectively, 20% for men and 21.9% for women [3, 4].

Although hypertension is a diffused pathology, it remains difficult to control. Only 60.6% of patients achieve BP control in Italy, and this control is better in < 70 years old females than in males of the same age [3]. Considering different areas of Italy, BP control is more efficient in the South (66.3%) compared to the Center (60.7%) and the North (55.6%) [5].

Similar results have been found in other countries. In Russia, BP control is achieved only in 52.9% of patients taking antihypertensive drugs; this percentage raises to 61.8 in Norway [6]. Comparable results were reported in the National Health and Nutrition Examination Survey (NHANES) 2017–2018, where 56.3% of US adults with hypertension had uncontrolled BP [7].

Resistant hypertension (RH) refers to patients with uncontrolled BP despite being treated with 3 different antihypertensive drugs at maximum tolerated dose, or to patients taking ≥ 4 medications, independently of BP control. Some factors, including younger age, male sex, obesity, longer duration of hypertension, and comorbidities correlate with an RH phenotype [8–11].

Another crucial aspect correlated with a poor BP control is the patients' consideration of their disease. It is estimated that 27.3% of patients with a diagnosis of hypertension do not monitor their BP [12].

With this observational study, we aimed to analyzing the prevalence of hypertension, uncontrolled hypertension and RH, and their association with risk factors or diseases, in a large cohort of patients referred to primary care physicians.

## Material and methods

We conducted a retrospective observational study to characterize the cohort of patients with arterial hypertension resident in Verona city and province (Italy), corresponding to approximately 900,000 individuals, referring, in the period when the study was conducted, to 524 general practitioners. In a population of 228,406 adults, we only collected data from people with a diagnosis of arterial hypertension for a total of 43,526 patients. To this purpose, we used the MySQL database, run by Azalea.NET, built on the medical records of 150 General Practitioners (GPs) who adhered to the databank for research purposes; each GP to be included in the study was required to take care of at least 700 patients. The data regarding BP control were taken by GPs during their daily clinical routine. The study was designed in 2020 and data were collected from January 1st 2016 to December 31 2017. The study was approved by the local Ethics Committee. Informed consent to participation was not required as all data were irreversibly anonymized. We selected data from patients with a diagnosis of hypertension: each diagnosis was made according to the ESC guidelines through the conventional office blood pressure measurement. After that, we classified the patient as hypertensive assigning him the specific ICD 9 code with the related medical exemption. Patient data included sex, age, BP values, number of prescribed pills and major cardiovascular comorbidities. To this scope, we consulted the database relative to the disease code ICD-9 for acute myocardial infarction, diabetes mellitus, chronic renal disease, heart failure and atrial fibrillation. The diagnosis of diabetes was based on plasma glucose criteria (plasma glucose > 126 mg/dL), or during a 75 g oral glucose tolerance test (2 h plasma glucose > 200 mg/dL), or glycated hemoglobin ≥ 6.5%, or symptomatic patient with random plasma glucose ≥ 200 mg/dL. Patients were classified as affected by chronic kidney disease if a glomerular filtrate rate (GFR) < 60 ml/min/1.73 m^2^ was present for > 3 months. The diagnosis of heart failure was based on symptoms and signs, clinical history and physical examination. The diagnosis of atrial fibrillation was based on rhythm documentation with an electrocardiogram.

Patients considered affected by RH were those with at least 1 BP measurement in the last 2 years that were treated with 3 different antihypertensive agents with recorded BP ≥ 140/90 mmHg, or patients taking ≥ 4 medications. In the group of patients taking 3 medications, the diuretic was not necessarily present [13] [14].

### Statistical analysis

Statistical analysis was conducted with Jamovi (ver. 2.3.18, The Jamovi project (2022 [Computer Software]. Retrieved from https://www.jamovi.org), Normality distribution of our variables was assessed with the Shapiro–Wilk’s test. For normally distributed variables, mean and the standard deviation is indicated. Pearson’s chi-squared test was used in the subgroup analysis for discrete variables. Student’s *t*-test or Mann–Whitney *U* test was used in subgroup analysis for continuous variables, according to normality, assessed with the Kolmogorov–Smirnov test. Logistic regression analysis was performed to define which variables were independent predictors of BP control. A *p*-value < 0.05 was considered as statistically significant. Images were elaborated with Microsoft Excel (Microsoft Corporation. (2018). Microsoft Excel. Retrieved from https://office.microsoft.com/excel).

## Results

Among the 228406 subjects included in the study, 43,526 had a confirmed diagnosis of arterial hypertension (19.06%). The characteristics of these patients are summarized in Table 1. Even though male patients were younger, they showed a significantly higher prevalence of all comorbidities. Instead, we did not observe differences in the number of antihypertensive drugs used.

**Table 1:** Characteristics of hypertensive patients stratified by gender

	Males *N* = 20,598	Females *n* = 22,928	*p-value*
Age *mean (IQR)*	60 (20)	70 (20)	**< 0.001**
Myocardial infarction *% (n)*	3.3 (682)	1.2 (278)	**< 0.001**
Diabetes *% (n)*	21.7 (4479)	17.2 (3936)	**< 0.001**
Chronic kidney disease *% (n)*	4.4 (912)	2.8 (638)	**< 0.001**
Heart failure *% (n)*	3.1 (641)	2.8 (631)	**0.026**
Atrial Fibrillation *% (n)*	10.6 (2179)	8.4 (1928)	**< 0.001**
Number of antihypertensive drugs *mean (IQR)*	2 (2)	2 (2)	n.s

Statistically significant values are reported in bold

Notably, 16,577 patients (38.09% of all hypertensive patients) did not have any BP measurement in the last 2 years, therefore, it is unknown whether their BP was controlled. Of the 26,949 patients with BP measurements in the last 2 years, 13,803 (51.2%) had controlled BP.

In the 26,949 patients, those with RH were 5620, with a prevalence of 20.85% in the hypertensive group and 2.46% considering the entire population. Of these patients with RH, 3,061 were already taking 4 medications, and 2559 were taking 3 medications with a recorded BP ≥ 140/90 mmHg.

### Drug utilization

**Fig. 1:**
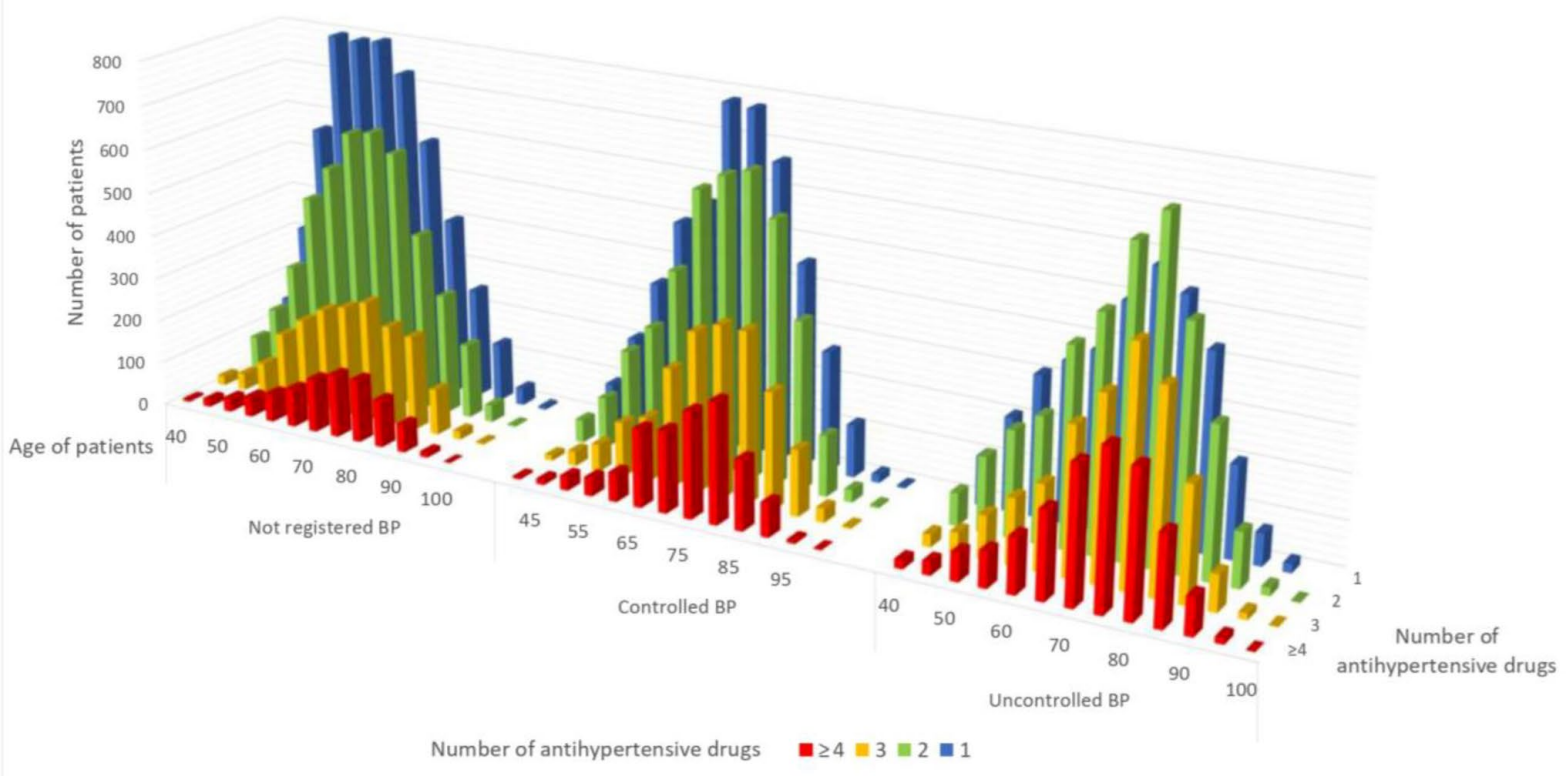
Number of antihypertensive drugs and their utilization in the different age groups

In the studied population, 5990 patients (13.76% of the hypertensive group) did not take any medication. In this population, we noticed that 1190 (19.87%) had good BP control, whereas 823 (13.74%) had poor control; the remaining 3977 (66.39%) did not have BP measurements in the last 2 years. Regarding this subgroup of patients, it may be hypothesized that those without pharmacological treatment were probably individuals initially subjected to lifestyle interventions or that refused taking medications. On the other side, patients with normal blood pressure, may have the benefit of some medical intervention, which we cannot determine because of the observational nature of this study. The number of antihypertensive drugs and their utilization in the different age groups are reported in Fig. 1. As it can be seen, when age increases, polytherapy becomes more frequent, at least up to 80 years of age, when an opposite trend starts to appear. Elderly patients (65 years or more) often required complex antihypertensive treatment (29% received 3–4 drugs) and their BP, despite a more stringent clinical follow-up (66% had BP recorded), was uncontrolled in 48% of cases. Patients without BP measurement in the last 2 years, had a mean age of 63.2 years and the average prescribed medications were 1.4.

### Blood pressure control and predictors of inadequate control

Comparison of the characteristics of patients with controlled and uncontrolled BP are summarized in Table 2***.*** The data show that 13,803 patients (31.71% of the hypertensive group) had good BP control, whereas 13,146 patients (30.20% of the hypertensive group) had poor BP control (≥ 140/90 mmHg). Age was not significantly different between the two groups.

**Table 2:** Clinical characteristics of hypertensive patients stratified by BP control

	Uncontrolled BP *n* = 13,146	Controlled BP *n* = 13,803	*p-value*
Sex, Female, *% (n)*	55 (7227)	53 (7313)	**0.001**
Age, *me. (IQR)*	70 (10)	70 (20)	n.s
Myocardial infarction, *% (n)*	2.0 (258)	2.5 (349)	**0.002**
Chronic kidney disease, *% (n)*	3.6 (475)	4.1 (565)	**0.041**
Diabetes, *% (n)*	25.1 (3303)	25.2 (3482)	n.s
Heart failure, *% (n)*	2.7 (352)	4.1 (565)	**< 0.001**
Atrial fibrillation, *% (n)*	9.1 (1194)	11.7 (1617)	**< 0.001**

Statistically significant values are reported in bold

**Fig. 2:**
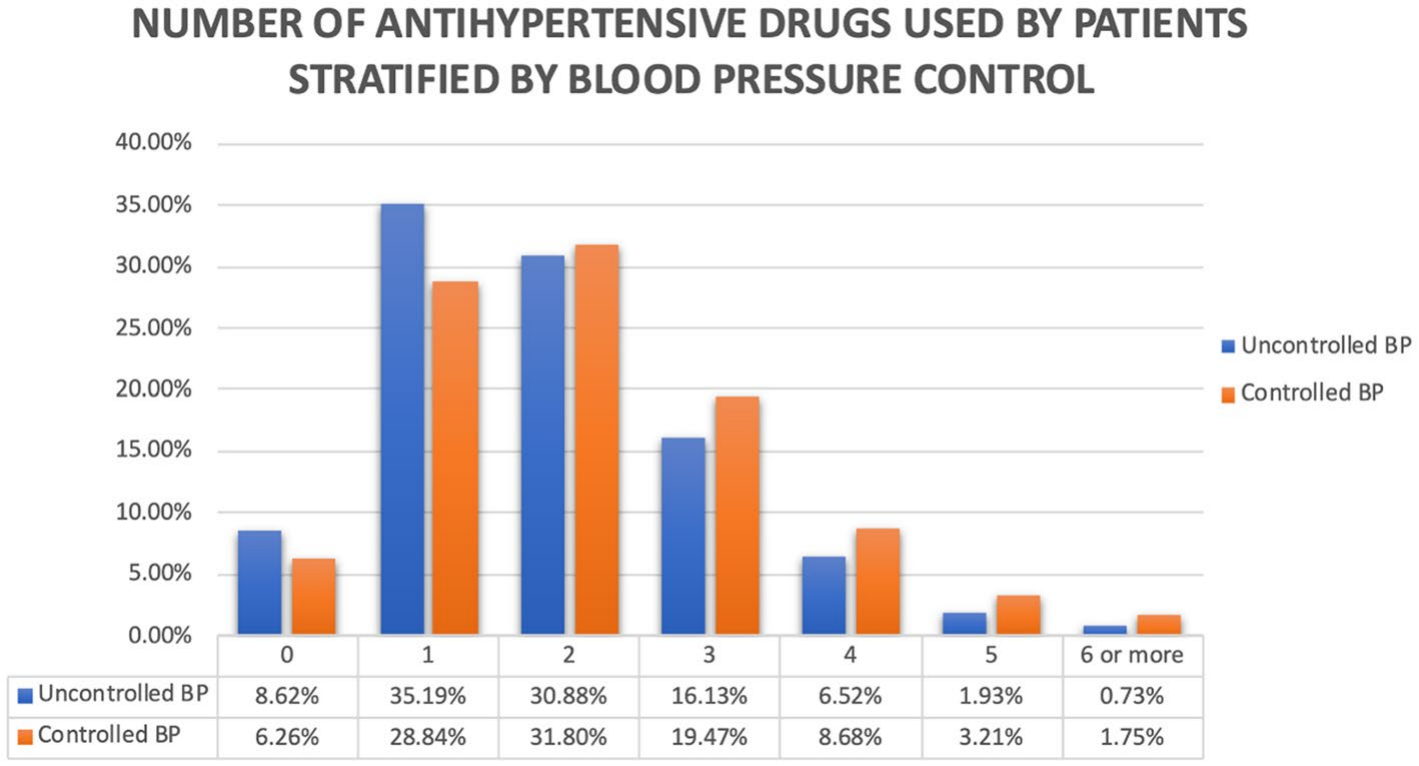
Number of antihypertensive drugs stratified by blood pressure control

As mentioned before, 16,577 patients did not have BP measurements done in the last 2 years (38.09% of the hypertensive group). Patients with inadequate BP control were mainly female. We hypothesize that they had a poorer BP control because of a lower occurrence of comorbidities. Patients with controlled hypertension used more antihypertensive drugs, as shown in Fig. 2. To determine which anamnestic factors were related to uncontrolled hypertension, we conducted an univariable analysis and then constructed a multivariable model, as shown in Table 3. By this analysis, we found that the female sex was associated with uncontrolled hypertension, whereas comorbidities (myocardial infarction, heart failure, and atrial fibrillation) were associated with good BP control.

**Table 3:** Univariable and multivariable models to assess the relationship between patient risk factors and uncontrolled blood pressure

	Univariable		Multivariable	
	OR (95%CI)	*p*-value	OR (95%CI)	*p*-value
Sex, female	1.08 (1.033–1.14)	**0.001**	1.07 (1.02–1.12)	**0.007**
Myocardial infarction	0.72 (0.66–0.91)	**0.002**	0.82 (0.7–0.97)	**0.018**
Diabetes		n.s		
Chronic kidney disease	0.88 (0.78–0.99)	**0.041**		n.s
Heart failure	0.64 (0.56–0.74)	**< 0.001**	0.71 (0.62–0.81)	**< 0.001**
Atrial fibrillation	0.75 (0.7–0.81)	**< 0.001**	0.79 (0.73–0.86)	**< 0.001**

Statistically significant values are reported in bold

### Resistant hypertension

We found 5620 patients with RH, with a prevalence of 20.85% in the hypertensive group and 2.46% in the entire population. Characteristics of these patients, compared with patients with non-resistant hypertension, are shown in Table 4. Patients with RH had a significantly higher prevalence of all considered comorbidities compared to those without RH. Furthermore, to find out what factors were related to RH, we conducted a univariable analysis and then constructed a multivariable model, as shown in Table 5. All the major comorbidities that we analyzed were associated with RH. Patients with uncontrolled RH were 4352. They were mainly female and showed a lower prevalence of all major cardiovascular comorbidities, similarly to what we found in the whole hypertensive population. We then performed an additional analysis in the group of uncontrolled RH patients to evaluate the associated comorbidities (Table 6). Female sex and all the major comorbidities that we analyzed were associated with the uncontrolled RH patients, similarly to what we found in the RH population.

**Table 4:** Clinical characteristics of the sample of patients with resistant hypertension

	Resistant *n* = 5620	Non-resistant *n* = 21,329	*p-*value
Female, *%(n)*	54.7 (3073)	53.8 (11,467)	n.s
Age *me. (IQR)*	70 (20)	70 (10)	**< 0.001**
Myocardial infarction, *%(n)*	4.1 (232)	1.8 (375)	**< 0.001**
Chronic kidney disease, *%(n)*	7.4 (414)	2.9 (626)	**< 0.001**
Diabetes, *%(n)*	34.4 (1931)	22.8 (4854)	**< 0.001**
Heart failure, *%(n)*	8.6 (486)	2 (431)	**< 0.001**
Atrial fibrillation, *%(n)*	19.6 (1100)	8 (1711)	**< 0.001**

Statistically significant values are reported in bold

**Table 5:** Univariable and multivariable models to assess the relationship between risk factors and resistant hypertension

	Univariable		Multivariable	
	OR (95%CI)	*p*-value	OR (95%CI)	*p*-value
Sex, female	1.04 (.98–1.01)	n.s	–	–
Myocardial infarction	2.41 (2.04–2.864)	**< 0.001**	1.94 (1.63–2.31)	**< 0.001**
Diabetes	1.77 (1.67–1.89)	**< 0.001**	1.63 (1.53–1.74)	**< 0.001**
Chronic kidney disease	2.6 (2.3–2.99)	**< 0.001**	1.86 (1.62–2.13)	**< 0.001**
Heart failure	4.59 (4.02–5.24)	**< 0.001**	2.98 (2.59–3.44)	**< 0.001**
Atrial fibrillation	2.79 (2.57–3.03)	**< 0.001**	2.3 (2.11–2.51)	**< 0.001**

Statistically significant values are reported in bold

**Table 6:** Univariable and multivariable models to assess the relationship between risk factors and uncontrolled resistant hypertension

	Univariable		Multivariable	
	OR (95%CI.)	*p*-value	OR (95%CI.)	*p*-value
Sex, female	1.32 (1.16–1.49)	**< 0.001**	1.20 (1.06–1.37)	**0.005**
Myocardial infarction	0.61 (0.46–0.81)	**< 0.001**	0.69 (0.52–0.93)	**0.014**
Diabetes	0.74 (0.65–0.84)	**< 0.001**	0.78 (0.68–0.89)	**< 0.001**
Chronic kidney disease	0.59 (0.47–0.73)	**< 0.001**	0.76 (0.61–0.96)	**0.021**
Heart failure	0.32 (0.26–0.38)	**< 0.001**	0.40 (0.33–0.49)	**< 0.001**
Atrial fibrillation	0.46 (0.40–0.53)	**< 0.001**	0.54 (0.47–0.63)	**< 0.001**

Statistically significant values are reported in bold

## Discussion

In this observational study, we examined a very large cohort of patients affected by arterial hypertension evaluating the prevalence, comorbidities and clinical features of the population.

Hypertension is a widespread pathology in Italy and worldwide. The knowledge of its prevalence and clinical features is very important and can be a powerful tool to improve its control, treatment and collaboration among patients, GPs and specialists.

The prevalence of hypertension in the studied population was 19.06%. This is consistent with our previous study conducted in a smaller cohort of patients (21.9%) [4], but it is lower than that estimated worldwide (31.1%) and in Italy (25.9%). This apparent discrepancy may be related to the number of undiagnosed hypertensive patients present in our cohort.

### Patients with uncontrolled and resistant hypertension

The present study shows that 20.85% of hypertensive patients had RH. This is in line with literature data reporting 5–30% RH prevalence, with a more likely prevalence of 10% [15–17]. Two American studies show a similar result: the first study analyzed 470386 hypertensive patients and showed a 15.3% RH prevalence; the other study conducted on more than 60,000 patients, showed a RH prevalence of 14.8% [18, 19].

It is a matter of concern that in a large number of hypertensive subjects, BP control is not achieved. In our study 13,146 patients (about 30% of the hypertensive group) did not achieve BP control, and, even worse, a very large number of hypertensive patients (16,577, 38% of the hypertensive group) did not have BP measurements done in the last 2 years. Therefore, we can assume that the prevalence of uncontrolled hypertension is underestimated. This evidence indicates that the management of these patients is not adequate, even though there might be some explanation. First of all, some BP measurements may be taken at home or in nursing homes and may not be registered in the GP database. Second, not all the GPs can be equally familiar with the use of an electronic database, therefore, some patient data were not reported. Finally, we should consider that some patients may measure BP by themselves at home without involving GPs.

A large group of patients with no BP records were those not taking anti-hypertensive drugs and with a very low overall number of taken medications (1.4). Considering the number of taken medications, it is not surprising that patients not taking medications or taking only one pill, were the ones that showed the worst blood pressure control (Fig. 2). In our cohort, 5990 patients were without therapy, and 66.39% (3977 patients) of them did not have BP recorded in the last 2 years. This number represents 13.76% of all patients, close to the 16.8% reported by the VII Report Health Search of the Research Institute of the Italian Society of General Medicine (SIMG) in 2012 [20].

This aspect may have some explanations. For instance, patients without therapy or taking a low number of medications can get out of GP sight and, therefore, do not receive proper assistance. Moreover, these patients may ignore their disease and do not measure their BP regularly.

Similar considerations can be proposed to explain the evidence that patients taking less medications are the ones with poorer BP control. Even in this case, the role of GPs is crucial to provide information, management and medical attention to establish a strong and fruitful doctor-patient alliance.

The analysis of BP control in other countries depicts an even worse scenario. Uncontrolled BP in North American people, aged between 40 and 59 years, is 41.7%, in individuals older than 60 years is 46%, and among people in the 20–39 year age range, 59.9% do not achieve an adequate BP control [21]. The SIIA (Italian Society of Arterial Hypertension) target for patients with controlled BP is 70%, which can be reached by implementing combination therapy and simplifying drugs intake with one only pill (SPC, single-pill combination) [22].

### Number of medications

In our study, stratifying drug utilization by age, we denoted an increase in the number of medications taken by the patients with the increase in age, at least until 80 years. This trend was reversed in patients > 80 years that assumed less drugs compared to patients ≤ 80 years. This can be explained by the prudent therapeutic approach toward frail people, justified by the need to limit drug-to-drug interactions in patients taking several drugs to treat multiple pathologies. The healthy survival effect may also justify such an approach. According to the latest European guidelines for pharmacological treatment strategies, to achieve BP control, combination therapy is preferred [1]. Consideration of monotherapy is useful in low-risk grade hypertension, in older patients (> 80 year) or frailer patients. A study made by Brigham University shows the number of drugs needed to achieve BP control. Considering men of all ages, 22.3% of patients needed one drug, 26.8% needed two drugs, 27.3% needed three drugs, 16.6% needed 4 drugs, and 7% of the analyzed group needed 5 drugs. In the female group, these percentages were, respectively, 11.5%, 19.6%, 28.9%, 23.7%, and 16.3%. [23]. In our cohort, 1244 patients (2.86% of hypertensive patients on pharmacological therapy) take 5 or more drugs, and 391 (0.9% of treated patients) take 6 or more drugs. However, it cannot be excluded that this is related to the presence of comorbidities, like myocardial infarction or heart failure, which are treated with drugs classified as antihypertensive.

### Factors associated with resistant and uncontrolled hypertension

Between 2005 and 2008 the NHANES study showed that 38% of hypertensive patients taking 2 or fewer drugs have uncontrolled BP; this cluster of patients is uncontrolled, but it does not enter the resistant group [24]. Yet, these two conditions partially overlapped. Particularly, approximately 8.4% and 17.4% of hypertensive patients were taking 3 or more drugs and did not achieve BP control [25–27]. In our multivariable analysis, we found that myocardial infarction, heart failure, and atrial fibrillation were associated with a higher probability of controlled hypertension (Table 3). This appears to be counterintuitive since in patients with less comorbidities, it should be easier to maintain better BP control; however, similar findings are reported by other studies [28–30]. A possible explanation can be that patients with important cardiovascular comorbidities are more closely monitored by physicians (both GPs and specialists) who adopt a more stringent BP target, thus using a higher number of antihypertensive drugs.

The awareness of the damage caused by hypertension is also relevant. It has been reported that higher awareness of hypertension-related damage and closer monitoring is associated with better hypertension control [31]. On the other hand, Del Pinto et al. found that BP status awareness rates were higher in patients with uncontrolled hypertension (85.1%) [32]. This apparent inconsistency may be explained by the fact that patients with a more severe form of hypertension tend to be more aware of their disease. At the same time, monitoring BP allows patients to better control their pathology. In patients with RH and with uncontrolled RH, we denoted a correlation with all the major cardiovascular comorbidities examined. In other words, all these comorbidities were associated with a higher probability to have RH and uncontrolled BP. This is consistent with what is already reported in the literature, in fact patients with RH have clinical characteristics consistent with a higher cardiovascular risk [10, 33].

## Study limitations

Mainly because of the huge cohort of patients, this study has several limitations.

First of all, this study is observational and possible medical changes, including therapy adjustments when uncontrolled BP is denoted are unknown. It is also unknown if some patients had white coat hypertension because this was not reported in the database, and 24 h BP monitoring was not available. Unfortunately, the data regarding the class of drugs and the maximally tolerated dose of anti-hypertensive drugs are lacking.

As highlighted in the results, a group of patients did not have any BP measurement done in the last two years, therefore, the prevalence of uncontrolled hypertension may be underestimated. Finally, we were not able to identify secondary causes of hypertension in the group of patients with resistant or uncontrolled hypertension.

## Conclusions

This study provides a real-life picture of a very large cohort of patients affected by arterial hypertension, uncontrolled hypertension and RH. This analysis shows that hypertension is an extremely widespread pathology and that a broad number of patients do not achieve adequate BP control. To improve the clinical management of these patients, it is very important to strengthen the collaboration between GPs and clinical specialists in hypertension. The present investigation may help to identify those patients who may benefit from active monitoring and intervention to improve their clinical outcomes. On the other hand, GPs should identify all patients with RH or uncontrolled hypertension and send them to the clinical specialist for further evaluation.
